# Demanding Diagnosis of Splenic Angiosarcoma as Cause of Delayed Treatment of Spontaneous Splenic Rupture: A Case Report and Literature Review

**DOI:** 10.1155/2017/6256102

**Published:** 2017-02-02

**Authors:** Sara Coppola, Andrea Leva, Fabio Pagni, Simone Famularo, Luca Gianotti

**Affiliations:** ^1^School of Medicine and Surgery, Department of Surgery, University of Milano-Bicocca, San Gerardo Hospital, Monza, Italy; ^2^Department of Pathology, University of Milano-Bicocca, San Gerardo Hospital, Monza, Italy

## Abstract

*Background.* Primary splenic angiosarcoma is a very rare mesenchymal malignant tumor associated with a poor prognosis due to its high metastatic potential. This disease can be easily neglected and spontaneous splenic rupture is a frequent manifestation at the time of diagnosis leading to a poor outcome because of peritoneal dissemination.* Case Presentation.* We describe the case of a 49-year-old man who presented with asthenia, left upper quadrant abdominal pain, and anemia. Computerized tomography scan showed an enlarged spleen with no nodules and a nontraumatic rupture of the splenic capsule. Splenectomy was performed on account of the severe anemia and histopathology examination showed a primary angiosarcoma.* Conclusions.* Splenic angiosarcoma should be considered as one of the differential diagnoses in patients with nontraumatic spleen rupture and a specific previous medical history. Regrettably, splenectomy allows for a definitive diagnosis but not a curative treatment.

## 1. Introduction

Primary angiosarcoma of the spleen is an extremely rare malignancy arising from the splenic sinusoidal vascular endothelium whose pathogenesis is still unknown.

It has been first described by Langhans in 1879 and the annual incidence ranges from 0.14 to 0.25 cases per million [1]. This aggressive disease usually presents in adults during their sixth and seventh decade [2]. Primary splenic angiosarcoma is associated with a very poor prognosis due to its difficultly of diagnosis and to its high metastatic potential. In fact, a mortality rate of 93% is reported within 29 months from diagnosis, regardless of treatment strategy because of dissemination [3, 4].

Splenectomy, prior to splenic rupture, is considered to be the only treatment that may result in long-term disease-free survival. Yet, this mesenchymal tumor can be easily neglected and splenic rupture with massive intra-abdominal hemorrhage is one of the most frequent manifestations at the time of diagnosis [2].

We report a case of this tumor and a systematic review of the available literature.

## 2. Case Report

A 49-year-old man was admitted to our emergency department at the beginning of January 2014 with left upper quadrant abdominal pain, recurrent macroscopic haematuria, and a first syncopal episode that occurred two days before. The pain was severe and dull and had started two hours prior to admission with no evident trigger factor. The same symptom had occurred one week before admission. The patient described fatigue, insomnia, severe weight loss, nocturnal sweating, and impotence occurring during the previous 4 months. These signs and symptoms were apparently not related to each other, and there was no evidence of related organic lesion that could explain them.

Hematologic, immunologic, and serologic tests were all negative, and still the patient symptoms worsened. In November 2013, the patient was hospitalized, for the first time, because of macroscopic haematuria. A left urethral stenosis was detected through an abdominal computed tomography (CT) scan that showed no intra- or extraluminal mass. Subsequently, an urethral stent was positioned. The CT scan also showed a moderately enlarged spleen (16 × 10 × 7 cm) without nodules. Autoimmunity screening and a complete panel of viral serology were performed with negative results. The second hospitalization occurred one month later because of right facial nerve paralysis. A brain CT scan, MRI, electromyography, and lumbar puncture were performed and were also negative. A spontaneous regression of the facial paralysis occurred within 10 days.

**Figure 1:**
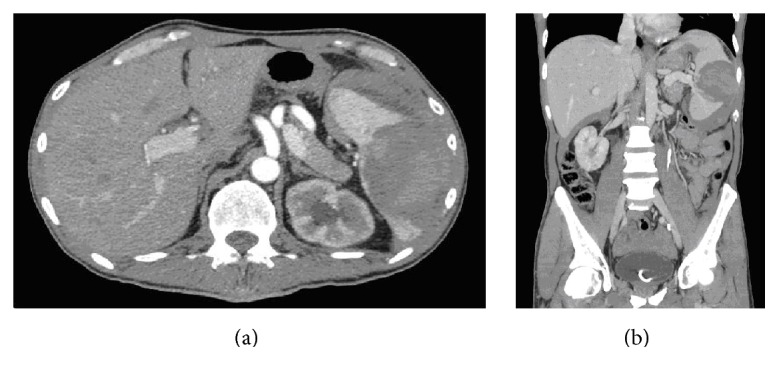
Axial CT scan demonstrating splenic bleeding (a); coronal scan (b).

At the moment of the last hospitalization, physical examination confirmed a splenomegaly. No enlargement of lymph nodes was detected. Anemia was present (hemoglobin: 8 g/L); CEA and CA19.9 were negative. An abdominal ultrasonography revealed hemoperitoneum and a splenic intraparenchymal hematoma. A subsequent CT scan of the whole abdomen confirmed the ultrasonography findings. The spleen was enlarged with an intraparenchymal hematoma (7 cm of diameter) with variable degrees of contrast enhancement as well as intra-abdominal free blood. Left kidney presented normal cortical thickness and the urethral stent was found to be in place (Figures 1(a) and 1(b)).

The doubt of a neoplastic process remained strong but still not proved. The patient was monitored over 24 hours, and, after a sudden decrease of hemoglobin levels (5 g/L), despite hemodynamic stability, the decision to perform a laparotomical splenectomy and nodal sampling was taken. Laparotomy showed hemoperitoneum (2 liters), splenomegaly with no active bleeding but with a 7 cm fissure of the splenic capsule at the upper pole. During the procedure, 3 units of packed red blood cells and 4 units of fresh frozen plasma were transfused. At macroscopical examination, a large hemorrhagic area was evident, while the spleen parenchyma had no nodular appearance. The general appearance of the surgical specimen was compatible with a nontraumatic hematoma or splenic fracture.

**Figure 2:**
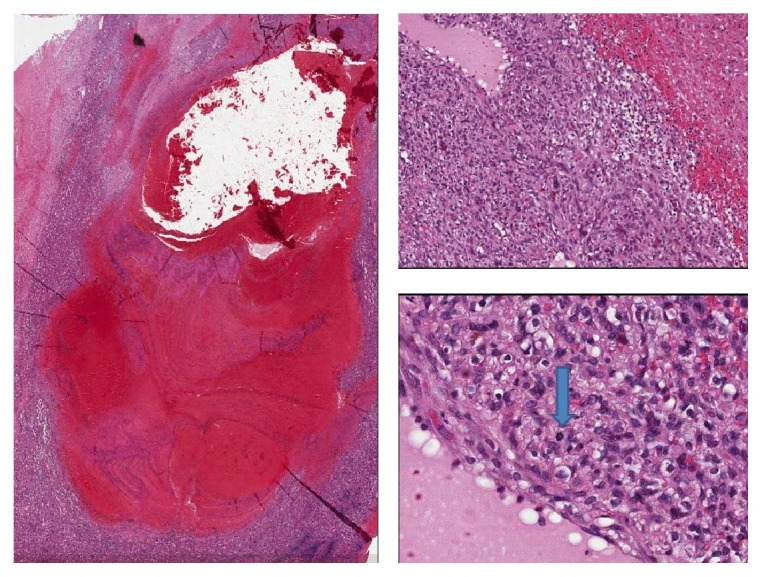
Histological findings: spleen angiosarcoma. At low-power field, histological sections revealed a large hemorrhagic areas with fibrin necrosis (H&E, 4x); at the periphery, foci of a solid vascular proliferation were evident (H&E, 10x). Spindle sarcomatoid cells with prominent hyperchromatic large elements characterized this high grade tumor. At high power field, atypical mitosis (arrow) and pseudovascular spaces were compatible with a final diagnosis of angiosarcoma (H&E, 40x).

However, at histological examination, the hemorrhagic lesion was found to be constituted by a central necrotic component with prevalent ischemic features but also rare coagulative aspects. At high power field, the microscopic examination revealed, at the margins of the necrotic core, the presence of residual vital areas constituted by spindle cells with an infiltrative pattern of growth into the normal red pulp. These mesenchymal cells showed moderate nuclear atypia and occasional mitoses consistent with a neoplastic nature (Figure 2). Immunohistochemistry showed strong reactivity for endothelial markers (CD34, factor VIII) and focal CD31 reactivity. Moreover, cytoplasmic positivity for CD68pgm1 confirmed a possible littoral origin of the neoplastic cells. Finally, the presence of clear cut tumoral necrosis was diagnostic of malignancy and suggestive of a primary splenic angiosarcoma.

The postoperative course was uneventful. The patient was referred to a specialized oncologic center for sarcoma. A second total body CT scan performed in February 2014 showed small peritoneal nodules which were confirmed by a PET scan.

A chemotherapy regimen with ifosfamide and doxorubicin was started and, after two cycles, the patient showed no further disease progression at CT and PET scan at a 3-month follow-up. Patient died in December 2014.

## 3. Discussion

Primary angiosarcoma of the spleen is a rare and aggressive soft tissue sarcoma. The poor prognosis of the disease is due both to its intrinsic malignant behavior with early metastatic spread and to the late diagnosis due to the unspecific presentation. Nontraumatic splenic rupture is a quite common clinical appearance (13–32%) and, at the same time, represents one of the worst prognostic factors because it may induce peritoneal and vascular dissemination [4].

As this case highlights, the unspecific clinical presentation of splenic angiosarcoma renders its diagnosis hard and delays treatment. Most common presenting symptoms are generic and variable and include weakness, fatigue, malaise, mild fever, and nonspecific abdominal pain (75–83%). Hemoperitoneum due to spontaneous splenic rupture is seen in up to 30% of cases and is often the first manifestation of the disease [12]. Anemia and thrombocytopenia are the most common laboratory abnormalities (75–100% and in 14–60% of cases, resp.) probably due to chronic disease, bleeding, hemolysis, and sequestration of erythroid elements and to a tumor-associated phenomena [4].

There is no defined radiologic element that can distinguish between angiosarcoma and other splenic lesions such as lymphoma, leukemia, or metastasis [7, 13]. For this reason, early diagnosis of splenic angiosarcoma is very difficult and definitive diagnosis is often achievable only postoperatively. Ultrasonography findings are variable and present a large spectrum ranging from mild splenomegaly to ill-defined solid and cystic masses with heterogeneous texture [14]. On contrast-enhanced CT scans, the tumor may exhibit peripheral or heterogeneous contrast enhancement similar to that of hepatic cavernous hemangiomas. Contrast enhancement MRI scan may show defined nodular lesions with high signal intensity related to subacute hemorrhage or tumor necrosis. Regardless of the selected technique, radiologic diagnosis of splenic angiosarcoma remains highly challenging due to the great overlap of characteristics with other vascular splenic tumors such as hemangiomas, littoral cell angiomas, lymphangiomas, hemangiopericytomas, and epithelioid vascular tumors [15]. For this reason, in our case, diagnosis was delayed and obtained only after splenectomy was performed because of the patient's life-threatening anemia.

Biopsy is contraindicated for splenic angiosarcoma because of the high risk of rupture. Therefore, histologic studies can only be made after splenectomy. Histological findings are similar to those of angiosarcoma seen in other parts of the body. Cut specimens usually reveal poorly defined nodular masses. Diffuse involvement is common, with replacement of the entire splenic parenchyma by the tumor. A solitary mass is a less common finding. Hemorrhage and necrosis are frequently seen within the tumor [4]. From the immunohistochemical point of view, at least two vascular proliferation markers (CD31, CD34, and factor VII) plus at least one histiocytic differentiation marker (lysozyme and/or CD 68) are required to make diagnosis [16].

Early diagnosis is crucial to allow a prompt treatment especially for highly malignant tumors to avoid local progression or metastatic dissemination. Montemayor and Caggiano, found that patients with splenic angiosarcoma had a longer survival if splenectomy was performed prior to rupture compared to after rupture (14.4 versus 4.4 months) suggesting that splenectomy should be carried out for all patients as early as possible not only for diagnostic purpose but also mainly for improving prognosis [17].

In our case, splenectomy was the only mean of diagnosis and it was performed after spleen fissure. Therefore, we might expect poor patients prognosis since the concomitant peritoneal dissemination was already present at the time of surgery.

Nevertheless, despite early splenectomy before rupture, the outcome of this disease remains poor, with only 20% of patients surviving after 6 months due to the very high frequency of metastasis. The metastatic rate is between 69% and 100% and the main metastatic sites are the liver (89%), lungs (78%), lymph nodes (56%), and bone (22%) [10]. Systemic adjuvant chemotherapy is only theoretically beneficial considering the early hematogenous micrometastatic potential of the disease. There are no recognized standard regimens. Some drugs, showing good efficacy for other soft tissue sarcomas, have been empirically employed for splenic angiosarcoma with poor results [18].

We reviewed literature and summarized the data regarding clinical presentations, laboratory results, mean of diagnosis, and treatment in Table 1. As described, signs and symptoms are absolutely generic and laboratory results (prevalently anemia and thrombocytopenia) may also suggest different unspecific hematologic disorders. In case of synchronous metastases, the diagnosis is easier to obtain through a radiologic workup because the neoplastic disease is evident and in general a nodular spleen is present. On the contrary, in our case, the first CT scan performed during the first hospitalization revealed only an enlarged spleen and the second one showed a spleen rupture in absence of nodules or masses. For these reasons, diagnosis was difficult to be achieved. The peculiarity of our case is in the panel of signs and symptoms spontaneously disappearing months before the splenic burst occurred. This scenario might be interpreted as part of a paraneoplastic syndrome. Although the suspicion of neoplasia was high, the tumor could not be diagnosed until surgery was required for bleeding.

## 4. Conclusion

In conclusion, splenic angiosarcoma should be taken into consideration as differential diagnosis in case of unexplained left upper quadrant abdominal pain, anemia, splenomegaly, and unspecific and self-recovering extra-abdominal signs and symptoms.

## Figures and Tables

**Table 1:** Review of literature.

Reference	Age	Clinical manifestations	Laboratory results	Radiologic Findings	Mean of diagnosis	Metastases at surgery	Treatment	Prognosis
Liu et al. 2012 [5]	33	Pain	NA	Splenic rupture	CT	Yes	Emergency splenectomy + CT	NA
Duan et al. 2013 [2]	65	Pain	Anemia	Splenic rupture	CT	Liver metastases	Emergency splenectomy	NA
Hamid et al. 2010 [6]	70	Dyspnea	Anemia	Left pleural effusion	CT	No	Elective splenectomy	AWD, 8 months
Oztürk et al. 2007 [7]	49	Pain	Anemia, thrombocytopenia	Nodular spleen, splenomegaly	CT, MRI	No	Elective splenectomy + CT	DOD, 7 months
Raffel et al. 2010 [8]	64	Fatigue	Thrombocytopenia	Nodular spleen, lytic bone lesions	CT biopsy (BOM neg)	Bone metastases	Elective splenectomy + CT	DOD, 4 months
Takeuchi et al. 2010 [9]	37	Pain	NA	splenomegaly	CT	No	Elective splenectomy	DOD, 24 months
Hara et al. 2010 [10]	48	Fatigue	Thrombocytopenia	Splenomegaly, hepatomegaly	CT, MRI, FDG-PET biopsy (BOM neg)	No	Elective splenectomy + CT	DOD, 72 months
Kranzfelder et al. 2012 [11]	62	Pain	NA	Splenic rupture, liver nodules	CT	Liver metastases	Emergency splenectomy + CT	DOD, 8 months

NA: not addressed; CT: computerized tomography; CHT: chemotherapy; DOD: date of death after diagnosis; MRI: magnetic resonance imaging; BOM: bone marrow biopsy.

## References

[1] Langhans T. (1879). Pulsating cavernous neoplasm of the spleen with metastatic nodules to the liver. *Archiv für Pathologische Anatomie und Physiologie und für Klinische Medicin*.

[2] Duan Y.-F., Jiang Y., Wu C.-X., Zhu F. (2013). Spontaneous rupture of primary splenic angiosarcoma: a case report and literature review. *World Journal of Surgical Oncology*.

[3] Falk S., Krishnan J., Meis J. M. (1993). Primary angiosarcoma of the spleen A Clinicopathologic Study of 40 cases. *American Journal of Surgical Pathology*.

[4] Neuhauser T. S., Derringer G. A., Thompson L. D. R. (2000). Splenic angiosarcoma: a clinicopathologic and immunophenotypic study of 28 cases. *Modern Pathology*.

[5] Liu Z., Du X., Li H. (2012). Primary splenic angiosarcoma. *Vasa*.

[6] Hamid K. S., Rodriguez J. A., Lairmore T. C. (2010). Primary splenic angiosarcoma. *Journal of the Society of Laparoendoscopic Surgeons*.

[7] Oztürk E., Mutlu H., Sönmez G., Sildiroğlu H. O. (2007). Primary angiosarcoma of the spleen. *The Turkish Journal of Gastroenterology*.

[8] Raffel S., Hildebrandt B., Grieser C., Pahl S., Sturm I. (2010). Thrombocytopenia as first manifestation of splenic angiosarcoma. *Annals of Hematology*.

[9] Takeuchi T., Iwasaki S., Miyazaki J. (2010). Matrix metalloproteinase-1 expression in splenic angiosarcoma metastasizing to the serous membrane. *International Journal of Clinical and Experimental Pathology*.

[10] Hara T., Tsurumi H., Kasahara S. (2010). Long-term survival of a patient with splenic angiosarcoma after resection, high-dose chemotherapy, and autologous peripheral blood stem cell transplantation. *Internal Medicine*.

[11] Kranzfelder M., Bauer M., Richter T. (2012). Littoral cell angioma and angiosarcoma of the spleen: report of two cases in siblings and review of the literature. *Journal of Gastrointestinal Surgery*.

[12] Maier A., Bataille F., Krenz D., Anthuber M. (2004). Angiosarcoma as a rare differential diagnosis in spontaneous rupture of the spleen. *Chirurg*.

[13] Ha H. K., Kim H. H., Kim B. K., Han J. K., Choi B. I. (1994). Primary angiosarcoma of the spleen CT and MR imaging. *Acta Radiologica*.

[14] Nahman B., Cunningham J. J. (1985). Sonography of splenic angiosarcoma. *Journal of Clinical Ultrasound*.

[15] Thompson W. M., Levy A. D., Aguilera N. S., Gorospe L., Abbott R. M. (2005). Angiosarcoma of the spleen: imaging characteristics in 12 patients. *Radiology*.

[16] McHugh M., Miettinen M. (1994). KP1 (CD68). Its limited specificity for histiocytic tumors. *Applied Immunohistochemistry & Molecular Morphology*.

[17] Montemayor P., Caggiano V. (1980). Primary hemangiosarcoma of the spleen associated with leukocytosis and abnormal spleen scan. *International Surgery*.

[18] Zwi L. J., Evans D. J., Wechsler A. L., Catovsky D. (1986). Splenic angiosarcoma following chemotherapy for follicular lymphoma. *Human Pathology*.

